# Effects of supplementing hen diet with *Lavandula angustifolia* and/or *Mentha spicata* essential oils on production performance, egg quality and blood variables of laying hens

**DOI:** 10.1002/vms3.343

**Published:** 2020-08-30

**Authors:** Mehran Torki, Ahmad Mohebbifar, Hamed Mohammadi

**Affiliations:** ^1^ Animal Science Department College of Agriculture and Natural Resources, Razi University Kermanshah Iran; ^2^ Department of Agriculture Payame Noor University Tehran Iran

**Keywords:** blood variables, egg production, herbal additives, laying performance, morphometric egg traits, phytogenic feed additives

## Abstract

**Background:**

Organic products of animals are getting more accepted by consumers. Using herbal additives may lead to more health animal products. In this research it is hypothesized that *Lavandula angustifolia* and/or *Mentha spicata* essential oils would be helpful to enhance production performance in laying hens.

**Objectives:**

This experiment was conducted to evaluate the effects of *Lavandula angustifolia* and *Mentha spicata* essential oils on performance, egg traits and blood variables in laying hens.

**Methods:**

144 Lohmann LSL‐Lite laying hens from 42 until 56 weeks of age were used in a completely randomized design in four treatments and six replicates (six birds per replicate). The treatments consisted of: (a) control group (basal diet), (b) basal diet supplemented with 250 mg/kg diet lavender essential oil (LEO), (c) basal diet supplemented with 250 mg/kg diet mint essential oil (MEO), and (d) basal diet supplemented with both LEO and MEO.

**Results:**

Using LEO and/or MEO did not affect body weight changes, feed intake, egg weight, egg index, yolk index, Haugh unit, egg shell weight and egg shell thickness. Feeding LEO, individually or in combination with MEO, did not affect FCR compared with the control group (*p* < .05), however, feeding MEO individually increased feed conversation ratio (FCR) compared to LEO and the control group during 42–56 weeks (*p* < .05), as well as decreasing egg mass compared to LEO (*p* < .05). Feeding LEO increased egg production compared to MEO and combination of MEO and LEO (*p* < .05).

**Conclusions:**

In conclusion, dietary supplemental MEO (250 mg/kg) may increase FCR, and LEO (250 mg/kg) is more effective than MEO (250 mg/kg) for egg production and egg mass purposes; besides MEO (250 mg/kg) negatively affected FCR compared with the control group. In addition, no specific beneficial effect of dietary supplemental MEO and/or LEO on the other measured variables was detected.

## Graphical abstract

Consumer pressure related to the potential development of antibiotic‐resistant bacteria has resulted in the development of non‐antibiotic feed additives that may also improve poultry performance. It is known that most of their properties are due to the essential oils and other secondary plant metabolites. Essential oils enhance production of digestive secretions, stimulate blood circulation, exert antioxidant properties, reduce levels of pathogenic bacteria and may enhance immune status. This paper shows that dietary supplemental *Lavandula angustifolia* essential oil have a favourable effect on egg production of laying hens compared to *Mentha spicata* essential oil or combined form.

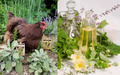

## 1 INTRODUCTION

The use of most antibiotic growth promoters has been banned in many countries, especially in the European Union since 2006 (Butaye et al., 2000). Herbal additives are given to animals or birds to improve their physiological and productive performance. The antimicrobial effect of some medicinal plants is reported (Valero & Salmeron, 2003). Medicinal plants, their extracts and essential oils have a wide range of activities, including inhibitory action on pathogens, effects on physio‐pathologies and activity in different body systems, e.g. endocrine and immune system (Francois, 2006). In layers and broilers, herbs and spices are not just appetite and digestion stimulants, but they can influence other physiological functions, help to sustain good health and welfare and also improve their performance (Frankic, Voljc, Salobir, & Rezar, 2009). The ajwain essential oil (*Trachyspermum ammi* L.) supplementation had a positive effect on the FCR, intestinal morphology and microbiota counts of breeder quails (Hajiaghapour & Rezaeipour, 2018).

The exact mechanisms by which plant components may alter blood profile are not fully understood; for instance, it is reported that peppermint (*Mentha piperita*) may prevent lipid peroxidation; besides, phenolic compounds and antioxidant activities found in peppermint tea may have hypoglycemic effects. It is also reported that peppermint (*Mentha piperita*) may interact with chromium resulting in insulin‐sensitive cell receptors or binding activity; in addition, some studies suggest that essential oils could increase insulin sensitivity‐receptors. The above‐mentioned mechanisms could lead to improve nutrient digestibility and modulate beneficial microbiota and also blood profile in laying hens' gut, which in turn may result in alter quantity and quality of their production. Nevertheless, exact mechanisms of action of phytogenic feed additives are still not fully understood.

*Lavandula angustifolia* (Common Lavender, English Lavender, French lavender, Garden Lavender, Lavender) is a flowering plant in the *Lamiaceae* family. Medicinal parts are usually roots, leaves or fruit of the plants, as well as essential oil from fresh flowers and/or the inflorescence; the flowers collected just before opening and dried, the fresh flowers and the dried flowers. Lavender oil has been reported to contain more than 100 components. The essential oil (1%–3%) of *Lavandula* is rich in linalool and linalyl acetate. Linalyl acetate is the major compound found in flowers. The plant contains also rosmarinic acid and coumarin (Lis‐Balchin, 2002). Carvacrol (26.2%), limonene (19.6%), 1,8‐cineole (11.8%), terpinen‐4‐ol (7.6%), spathulenol (4.9%), α‐pinene (4.2%), p‐cymene (4.2%), caryophyllene oxide (2.7%) and terpinolene (2.6%) were detected in *Lavandula angustifolia* essential oil by Bakhsha, Mazandarani, Aryaei, Jafari, & Bayate, 2014.

*Mentha spicata* (Spearmint), belongs to the *Lamiaceae* family. The plants of this family are a rich source of polyphenols, flavonoids and carvone, thus possessing strong antioxidant properties (Bimakr et al., 2011; Gulluce et al., 2007). The broiler chicks fed diet supplemented with of 2% spearmint was lower significantly in the serum total cholesterol compared with the control group and other groups (Abu Isha, El‐Hamid, Ziena, & Ahmed, 2018). It is stated that the improvement of FCR resulted from the increase in appetite due to the stimulation of salivary and gastric glands by spearmint oil, the decrease in pathogenic bacteria and better digestibility; besides, it is suggested that spearmint oil may stimulate salivary and gastric glands, and decrease bacteria which in turn improve digestibility and FCR (Abu Isha et al., 2018 qouted from Amal, 2012). Abu Isha et al., (2018) reported that, the addition of spearmint essential oil, to the diet increased significantly the feed intake of broiler chicks (Abu Isha et al., 2018). In addition, it is reported that feeding spearmint increases feed intake and consequently improves growth in broiler chickens (Saleh, Ijiri, & Ohtsuka, 2014). Galib, Al‐kassi, and Noor (2010) stated that the broiler chicks fed on peppermint (Mentha piperita) powder consumed significantly more feed consumption compared to the control group.

On this basis, we hypothesized that inclusion of *Lavandula angustifolia* and/or *Mentha spicata* into laying hens' diets would be helpful to enhance production performance, egg traits and blood biochemical variables; besides, assessing the probable synergistic interaction between dietary *Lavandula angustifolia* and *Mentha spicata* can be mentioned as novelty of the current study.

## 2 MATERIALS AND METHODS

### 2.1 Laboratory animals, experimental design and treatments

All experimental protocols were adhered and were approved by the guidelines of the Animal Ethics Committee of Razi University, Kermanshah, Iran (The animal use protocol number: Anim Sci, 116, Sep 2017). Hundred and forty four Lohmann LSL‐Lite laying hens in Phase II (42 weeks of age) were weighed individually and randomly assigned to four treatments with six replicates and six birds in each replicate in a completely randomized design. Four dietary isocaloric and isonitrogenous diets were used during three experimental periods, 42–46, 47–51 and 52–56 weeks. An empty cage was kept between treatments to eliminate cross‐feeding. The birds were placed in the cages and kept under 16 hr of light/8 hr dark cycle during the entire experimental period (14 weeks). The birds were housed under temperature 18–20°C for 14 weeks. Average ambient relative humidity inside the rearing house was 40%. The feed was offered on the basis of the recommendations (110 g/hen/day), and water (18 ± 4°C) was supplied ad‐libitum. To meet nutritional requirements of laying hens as recommended by the Lohmann LSL‐Lite catalogue (Lohmann LSL‐Classic International 2011), a corn‐soybean meal basal diet, 14.69% crude protein and 2,750 kilocalories of metabolizable energy per kg of feed was formulated, since we tried to maintain constant ME:CP ratio (Table 1).

**TABLE 1:** Ingredients and nutrient composition of the basal diet (%, unless stated otherwise)

Ingredients	Treatment 1 (control group)	Treatment 2	Treatment 3	Treatment 4
Corn	67.6	67.6	67.6	67.6
Soybean meal (44% CP)	21	21	21	21
Wheat bran	0.15	0.15	0.15	0.15
Soybean oil	0.07	0.07	0.07	0.07
Lime stone	3	3	3	3
Oyster shells	5.47	5.47	5.47	5.47
Dicalcium phosphate	1.64	1.64	1.64	1.64
NaHCO_3_	0.18	0.18	0.18	0.18
Common salt	0.19	0.19	0.19	0.19
Min. premix^a^	0.25	0.25	0.25	0.25
Vit. premix^b^	0.25	0.25	0.25	0.25
DL‐methionine	0.15	0.15	0.15	0.15
Lavender essential oil	0	0.025	0	0.025
Mint essential oil	0	0	0.025	0.025
Nutrient composition (as fed basis)				
2,750	ME (Kcal/kg)			
14.7	Crude protein			
3.64	Calcium			
0.37	Available phosphorus			
0.15	Sodium			
2.32	Crude fiber			
207	(Na + K)‐Cl (meg/kg)			
0.71	Lysine			
0.37	Methionine			
0.63	Methionine + cystine			
0.54	Threonine			
0.16	Tryptophan			

^a^ Vitamin mixture per 2.5 kg of diet provides the following: vitamin A, 7,700,000 IU; vitamin D_3_, 3,300,000 IU; vitamin E, 6,600 mg; vitamin K_3_, 550 mg; thiamine, 2,200 mg; riboflavin, 4,400 mg; vitamin B6, 4,400 mg; capantothenate, 550 mg; nicotinic acid, 200 mg; folic acid, 110 mg; choline chloride, 275,000 mg; biotin, 55 mg; vitamin B_12_, 8. 8 mg.

^b^ Mineral mixture per 2.5 kg of diet provides the following: Mn, 66,000 mg; Zn, 66,000 mg; Fe, 33,000 mg; Cu, 8,800 mg; Se, 300 mg; I, 900 mg.

Supplemental *Lavandula angustifolia* and *Mentha spicata* essential oils were purchased from Barij Essence Pharmaceutical Co, Kashan, Iran; their active ingredients, according to the information received from the manufacture, are presented in the Table 2. Accordingly, 250 mg/kg diet LEO provided the followings per kg diet: 1 4.53 mg limonene, 69.75 mg 1,8‐cineole, 18.10 mg Camphor, 76.00 mg linalool, 3.15 mg linalyl acetate, and 1.05 Terpinen‐4‐ol; and 250 mg/kg diet MEO provided the followings per kg diet: 3.15 mg 1,8‐Cineole, 70.25 mg limonene, 5.60 mg menthol, 3.95 mg pulegone, 139.50 mg carvone, and 0.78 mg menthone. To minimize probable essential oil evaporation, the feeds were mixed weekly with the additives and kept in tied, double layered plastic bags in a dry, dark and well‐ventilated room at 25°C (Attia, Bakhashwain, & Bertu, 2017). The birds received one of the four experimental diets including: (a) the basal diet (control group), (b) basal diet supplemented with 250 mg/kg lavender essential oil (LEO), (c) basal diet supplemented with 250 mg/kg mint essential oil (MEO), and (d) basal diet supplemented with 250 mg/kg lavender +250 mg/kg mint essential oil. The levels of the essential oils used in this experiment were chosen based on previous results reported by Abd El‐Motaal, Ahmed, Bahakaim, and Fathi (2008), Hassan, El Sanhoury, Ali, and Ahmed (2011) and Akbari and Torki (2014).

**TABLE 2:** The active ingredients of lavender and mint essential oils used in the current experiment

Name	Compound	(%)
Lavender essential oil	Limonene	1.81
	1,8‐Cineole	27.9
	Campher	7.24
	Linalool	30.4
	Linalyl acetate	1.26
	Terpinen−4‐ol	0.420
Mint essential oil	1,8‐Cineole	1.26
	Limonene	28.1
	Menthol	2.24
	Pulegone	1.58
	Carvone	55.8
	Menthone	0.310

### 2.2 Productive performance and egg quality

The productive performance of the laying hens including hen‐day egg production, feed intake and egg weight were recorded daily, and feed conversation ratio (FCR) and egg mass were calculated. To evaluate egg quality characteristics including egg quality traits yolk colour, egg shell thickness, egg shell weight, egg index, yolk index and Haugh unit (HU), egg samples were collected twice on week 7 of experiment and each time all eggs during three frequent days were used. The eggs were marked according to the diet and replicate group.

Haugh units were calculated from the records of albumen height and egg weight using the HU formula (Eisen, Bohren, & McKean, 1962). The shell thickness was a mean value of measurement at three locations on the egg (air cell, equator and sharp end) by using a dial and pipe gage. The yolk colour was scored with the aid of Roche Yolk Colour Fan. Yolk height and yolk diameter were measured by a tripod micrometer (Mitutoyo, 0.01 mm) and compass (Swordfish, 0.02 mm), respectively, then the yolk index was calculated. Egg length and width were individually recorded and used to calculate egg shape index.

### 2.3 Blood biochemical variables

At the end of the experiment, eight hens were randomly selected from each treatment and blood samples were collected from the brachial vein into a 5‐ml syringe. The collected blood samples were centrifuged at 805 *g* for 10 min, and the sera was frozen at −20°C until the analysis. Serum samples were thawed at room temperature and were analysed for glucose, triglyceride, albumin, uric acid and cholesterol according to the manufacture recommendations (Pars Azmun); besides, the enzyme activity of plasma glutathione peroxidase was measured according to the manufacture recommendations (Randox Laboratories kit).

### 2.4 Statistical analysis

**FIGURE 1:**
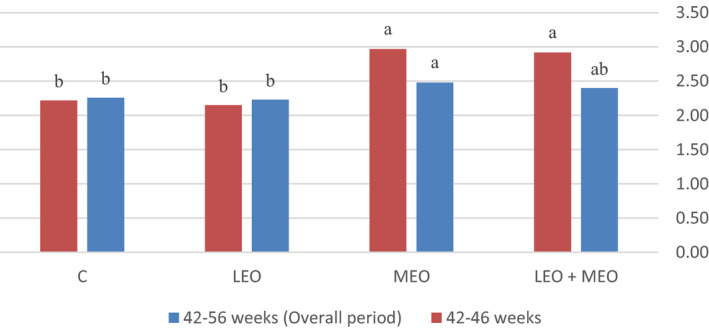
Effects of dietary treatments on feed conversion ratio (feed/egg) of the laying hens

**FIGURE 2:**
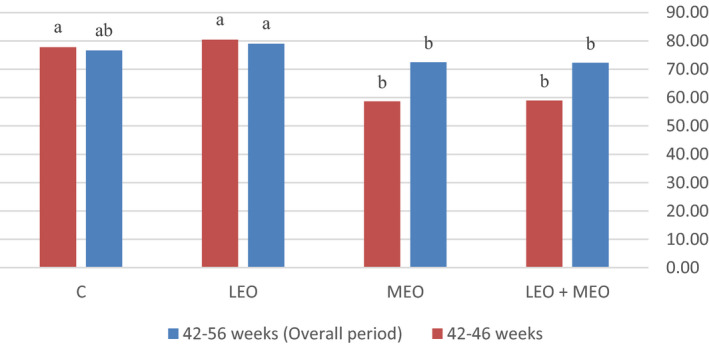
Effects of dietary treatments on hen‐day egg production (%) of the laying hens

**FIGURE 3:**
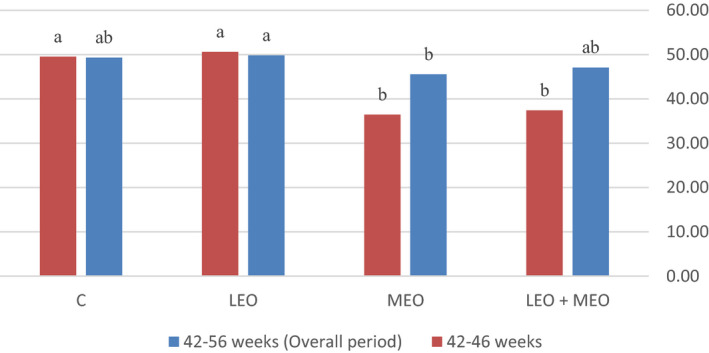
Effects of dietary treatments on egg mass (g hen^−1^ day^−1^) of the laying hens

All data obtained from the trials were subjected to the analysis of variance procedure of statistical analysis system (SAS, 2001, Version 8.02) according to completely randomized design. Means were separated by Duncan's new multiple range test. The level of significance was determined at <.05. The following model was considered for analysis: Y_ij_ = μ + (R_i_) + (C_j_) + (RC_ij_) + (e_ij_), where Y_ijk_ is the measured characteristic, μ is the overall mean, (R_i_) is the main effect of LEO, (C_j_) is the main effect of MEO, RC_ij_ is interaction between the effect of LEO and MEO, and (e_ij_) is the residual error. The effects of the main factors were not considered, whenever the interaction was significant.(Figures 1, 2, 3).

## 3 RESULTS

### 3.1 Production performance

The productivity data of laying hens are summarized in Table 3. The result of the present study showed that adding LEO and/or MEO essential oils did not affect feed intake and body weight changes during the experimental period (*p* > .05). In this research feeding LEO, individually or in combination with MEO, did not affect FCR compared with the control group (*p* < .05), but feeding MEO individually increased FCR compared with LEO and the control group during 42–56 weeks (*p* < .05); besides, feeding MEO individually or in combination with MEO increased FCR compared with LEO and the control group during 42–46 weeks (*p* < .05). In the present study, supplementation of MEO individually or in combination with LEO significantly decreased egg production and also egg mass values during 42–46 weeks (*p* < .05). Egg production was significantly increased due to LEO feeding compared to MEO and LEO + MEO groups during 42–56 weeks (*p* < .05). LEO feeding also increased egg mass compared to MEO group during 42–56 weeks (*p* < .05).

**TABLE 3:** Effects of dietary treatments on production performance of the laying hens

Productive performance	Treatments				SEM^a^	*p*‐values
	C	LEO	MEO	LEO + MEO		
Body weight changes (g)						
52–56 weeks	55.9	52.1	49.7	59.0	32.07	.167
Feed intake (g/hen/day)						
42–46 weeks	110	108	108	109	0.627	.362
47–51 weeks	109	109	109	109	0.195	.574
52–56 weeks	109	109	109	108	0.510	.609
42–56 weeks (overall period)	109	109	109	109	0.350	.576
Feed conversion ratio (feed/egg)						
42–46 weeks	2.22^b^	2.15^b^	2.97^a^	2.92^a^	0.050	.001
47–51 weeks	2.55	2.59	2.55	2.44	0.094	.732
52–56 weeks	2.01	1.93	1.93	1.85	0.072	.471
42–56 weeks (overall period)	2.26^b^	2.23^b^	2.48^a^	2.40^a^	0.059	.022
Hen‐day egg production (%)						
42–46 weeks	77.8^a^	80.4^a^	58.7^b^	59.0^b^	1.43	.001
47–51 weeks	67.0	67.1	67.8	68.1	2.12	.977
52–56 weeks	85.0	89.6	91.1	89.9	2.92	.491
42–56 weeks (overall period)	76.6^a^	79.0^a^	72.5^b^	72.3^b^	1.79	.040
Egg mass (g/hen/day)						
42–46 weeks	49.5^a^	50.61^a^	36.5^b^	37.4^b^	0.969	.001
47–51 weeks	43.5	42.40	43.4	45.0	1.48	.678
52–56 weeks	54.9	56.50	56.7	58.8	1.89	.549
42–56 weeks (overall period)	49.3^a^	49.8^a^	45.5^b^	47.1^a^	1.24	.077

Note

Means within a row with different superscripts are significantly different (*p* < .05).

Abbreviations: C, control group; LEO + MEO, lavender essential oil + mint essential oil; LEO, lavender essential oil; MEO, mint essential oil.

^a^ Standard error of mean.

### 3.2 Egg quality traits

Supplementation of the layer diet with LEO and/or MEO had no significant effects on egg weight, egg index, yolk index, HU, yolk colour, egg shell weight and egg shell thickness (*p* > .05) (Table 4).

**TABLE 4:** Effects of dietary treatments on egg quality traits of the laying hens

	Variables							
Treatments	Egg weight (g)	Egg index (%)	Yolk index (%)	Haugh unit	Yolk colour	Egg shell weight (g)	Egg shell thickness (mm 10^−2^)	
Control	65.5	73.7	40.5	77.3	6.83	5.91	37.3	
LEO	62.5	71.7	41.4	79.6	7.00	5.93	38.0	
MEO	62.5	72.7	40.0	77.6	6.58	5.72	37.7	
LEO + MEO	63.1	74.4	39.9	75.9	6.91	6.05	39.1	
SEM^a^	1.44	18.2	0.859	1.89	0.120	0.163	0.67	
*p*‐values	.424	.382	.604	.586	.117	.559		.311

Abbreviations: C, control group; LEO + MEO, lavender essential oil + mint essential oil; LEO, lavender essential oil; MEO, mint essential oil.

^a^ *SEM*.

### 3.3 Blood metabolites

Table 5 shows data obtained on the effect of experimental treatments on blood variables. No significant influence of experimental diets on albumin, uric acid, glucose, triglyceride and cholesterol was observed (*p* > .05).

**TABLE 5:** Effects of dietary treatments on biochemical variables of the laying hens

	Variables				
Treatments	Albumin (g/dl)	Uric acid (mg/dl)	Glucose (mg/dl)	Triglycerides (mg/dl)	Cholesterol (mg/dl)
Control	7.17	77.2	975	847	723
LEO	6.76	78.3	850	833	695
MEO	6.38	79.2	855	861	998
LEO + MEO	6.81	79.2	915	805	972
SEM^a^	0.443	23.4	86.1	30.1	242
*p*‐values	.671	.635	.709	.600	.730

Abbreviations: C, control group; LEO + MEO, lavender essential oil + mint essential oil; LEO, lavender essential oil; MEO, mint essential oil.

^a^ *SEM*.

## 4 DISCUSSION

### 4.1 Production performance

Several medicinal plants (e.g., garlic, onion, thyme, spearmint, pepper, yucca, black cumin (black seed), ginger) have been used in animal and poultry (layers and broilers) production due to their health benefits, e.g., spearmint leaves is used as carminative, antispasmodic and choleretic in human reported by Al‐Kassie (2010). It is hypothesized that herb mixture (garlic, onion, thyme, spearmint, pepper, yucca, black cumin seed, and ginger) might have several vital benefits of such as antioxidant, antimicrobial and anti‐inflammatory, which raise its use as herbal feed additives as alternative to antibiotics in broilers diets (Saleh, Ebeid, & Abudabos, 2018). The ajwain essential oil (*Trachyspermum ammi* L.) supplementation did not affect egg weight, egg mass, egg production, and feed intake in quail breeders (Hajiaghapour & Rezaeipour, 2018). Spearmint is used as an anti‐spasmodic, choleretic and carminative (Al‐Kassie, 2010). It is reported that, addition of spearmint essential oils, to the diet increased significantly the feed intake of broiler chicks (Abu Isha et al., 2018), Feeding spearmint increases feed intake and consequently improves growth in broiler chicks (Saleh et al., 2014).

Some aromatic herbal essences like lavender contain phytoestrogens. According to Saleh, Ahmed, and Ebeid (2019) inclusion of mixed sources of phytoestrogens (combination of 1 g/kg flaxseeds and 1 g/kg fenugreek) in diets improve laying performance, egg quality, the antioxidative status, hormonal profile and steroidogenesis in aged laying hens. Generally it is assumed that essential oils of herbs and spices might improve the palatability of feed due to their flavourful characteristics, hence could promote feed intake when added to layers and broilers diets (Hertrampf, 2001; Williams & Losa, 2001).

In this study, the addition of LEO and MEO essential oils did not influence feed intake and body weight changes during the trial period. In agreement, Nasiri‐Moghaddam, Hassanabadi, and Bidar (2012) reported that there was no difference in feed intake values between broilers fed control and those fed lavender essence (350 mg/kg) at the period of 22–42 days age.

The feed conversion ratio (FCR) describes the hens’ overall efficiency in converting ingested feed mass into egg mass over a specific period of time. An important claim often made for phytogenic feed additives is improvement of the FCR and thereby enhancing the intestinal availability of essential nutrients for absorption (Langhout, 2000; Williams & Losa, 2001). In this research feeding LEO, individually or combined with MEO, did not influence FCR compared with the control, but feeding MEO individually significantly increased FCR compared with LEO and control group during 42–56 weeks. In contrast, Nasiri‐Moghaddam et al., (2012) reported that dietary lavender essential oil (at 350 mg/kg) significantly improved FCR in broilers. In addition, Salari et al reported that feeding *Lavander stoechas* essence (200, 400 and 600 ppm) caused insignificant improvement in FCR in laying hens (Salari, Taki, Bojarpour, Sari, & Taghizadeh, 2014).

Isoprene derivatives, flavonoids, glucosinolates and other plant metabolites may affect the physiological and chemical function of the digestive tract. The stabilizing effect on intestinal microbiota may be associated with intermediate nutrient metabolism (Baratta, Dorman, Deans, Biondi, & Ruberto, 1998; Horton, Fennell, & Prasad, 1991; Jamroz et al., 2003). The pharmacological action of active plant substances or herbal extracts in humans is well‐known, but in animal nutrition the number of precise experiments is relatively low.

In this study, feed intake and body weight changes were not affected by supplemental MEO but FCR was affected by the supplementation of MEO compared to LEO and the control group. Body weight is not a common performance criteria in laying hens since hens are rather selected for laying performance and efficiency of feed conversion per unit of egg mass output. In a number of trials, laying rate, feed intake and FCR were not changed. Several studies confirmed the positive influence of herbs and their respective essential oils on body weight of hens with altered production performance (Bozkurt, Alcicek, Cabuk, Kucukyilmaz, & Catli, 2009).

In an experiment curcumin (0.5 g), capsaicin (0.015 g), piperine (0.02 g), ginger (0.05 g), cumin (1.25 g), asafoetida (0.25 g), ajwan (0.2 g), fennel (0.5 g), coriander (2.0 g), mint (1.0 g), garlic (0.5 g) and onion (2.0 g) could shorten the time of feed passage through digestive tract in rats (Platel & Srinivasan, 2001). Herbs with growth promoting activity increase the stability of feed and beneficially influence the gastrointestinal ecosystem mostly through growth inhibition of pathogenic microorganisms’ growth (Windisch, Schedle, Plitzner, & Kroismayr, 2008). Therefore, it might be possible that the increase in digestion and absorption of essential nutrients due to increasing the enzyme activity and/ or inhibition of pathogenic microorganism's growth could be the main reason of medicinal plants to accelerate the performance.

In a research, Al‐Ankari, Zaki and Al‐Sutan found that supplementation of *Mentha piperita* at the level of 1.5% for 35 days showed beneficial results on body weight, feed intake and FCR in broilers (Al‐Ankari, Zaki, & Al‐Sutan, 2004). Similarly, Nobakht, Norany & Safamehr reported that feeding 0.5% dried *Mentha pulegium* resulted in positive effects on performance in broilers at 42 days of age (Nobakht, Norany, & Safamehr, 2011). Whereas, Ocak et al. reported that supplemental *Mentha piperita* (0.2%) had no effects on broilers body weight and FCR at 42 days of age (Ocak et al., 2008). The controversy among these studies might be due to the differences in mint resources, which vary according to species, active ingredient, harvest time, etc (Brenes & Roura, 2010).

The phytogenic feed additives as performance enhancers for laying hens have primarily been supplemented to increase the utilization of the limit‐fed diet and, in turn, improve egg production (Bozkurt et al., 2009). In the current study, supplementing MEO individually or combined with LEO significantly decreased egg production and also egg mass values during 42–46 weeks; in addition, egg weights in MEO and LEO groups were not influenced compared to the control group. Egg production was significantly higher in LEO group compared to MEO and LEO + MEO groups during 42–56 weeks. LEO feeding also significantly increased egg mass compared to MEO group during 42–56 weeks. According to some authors, diets with higher inclusion levels of essential oils compounds had toxic effects (Krishan, & Narang, 2014). In this case, decline in egg production in treatment 4 (LEO + MEO), may be due to the accumulation of the essential oils and its probable toxicity; however, more adequate toxicological study must be carried out to clarify this assumption.

The results of this experiment are in accordance with the results of Nasiri‐Moghaddam, Hassanabadi & Bidar who did not observe any increases in egg weight, feed intake and egg production percentage by using lavender essence in the diets of layers (Nasiri‐Moghaddam et al., 2012). In addition, Bozkurt et al. showed that supplemented diets with mixture of essential oil (i.e., oregano oil, laurel leaf oil, sage leaf oil, myrtle leaf oil, fennel seed oil and citrus peel oil) (24 and 48 mg/kg of diet) did not affect egg production and egg weight of broiler breeders (Bozkurt et al., 2009). In another study (Botsoglou et al., 2005), the effects of dietary aromatic plant extracts (rosemary at 5 g/kg diet, oregano at 5 g/kg diet and saffron at 20 mg/kg diet) on the laying performance of hens from 32 to 40 weeks of age were investigated and the results showed no significant differences in egg production and egg weight among the treatment groups.

These observations partially support the hypothesis that herbs and their associated essential oils may favourably affect hen performance but the number of experimental studies conducted under standardized field conditions with larger numbers of hens and for longer durations, preferably over a whole laying cycle, is still limited (Bozkurt et al., 2009). In the current research, the essential oils did not increase egg production and egg mass, but as numerical, the highest egg production percentage and egg mass were found in LEO group.

Most of findings showed that improving FCR was related to increase in the amount of egg mass; consequently, increase in egg mass might lead to improved FCR (Nobakht et al., 2011; Sayedpiran, Nobakht, & Khodaei, 2011). In a study, the impact of feeding different amounts of eucalyptus (eucalyptus is similar to lavender due to containing cisteol) on productive performance, egg quality, blood variables and immune response in Japanese quails was investigated and it indicated enhanced daily weight gain, number of egg, egg weight, egg mass, improved FCR and quality of egg in the diets with eucalyptus compared to the control diet (Hassan et al., 2011). The results of our experiment are not in accordance with the results of Hassan, et al. that observed increase in egg weight, feed intake and production percentage by using eucalyptus powder in the diets of quails (Hassan et al., 2011). In another study, applying 3 g of eucalyptus per kilogram of diet improved the laying hens’ productive performance and immunocompetence; moreover, FCR was improved in the hens given 3 g of eucalyptus in the diet because of their health and vitality (Abd El‐Motaal et al., 2008). Different medicinal herbs contain different percentage of constituents and probably for this reason have different effects on productive performance.

### 4.2 Egg quality traits

Supplementation of the layer diet with *Lavandula angustifolia* and *Mentha spicata* essential oils had no significant effects on egg weight, egg index, yolk index, HU, yolk colour and egg quality variables (egg weight and egg shell thickness). The results of this study are in agreement with those reported by Salari et al. who stated that feeding lavender essence (200, 400 and 600 ppm) did not affect HU (Salari et al., 2014). It was observed from this study that feeding MEO did not increase egg weight. Increased egg weight might be due to better utilization of nutrients by mint, which in turn resulted in better egg weight (Merina Devi, Palod, Dar, & Shekhar, 2018). The results of the present study are not in line with the findings of Abdel‐Wareth and Lohakare, who reported that supplementation of peppermint leaves (5, 10, 15 or 20 g/kg) in laying hens improved egg weight due to the beneficial action of peppermint in the process of oviposition and imperative effect on the conversion of digested feed into eggs (Abdel‐Wareth & Lohakare, 2014). Dietary MEO supplementation did not influence HU which is not in agreement with findings were recorded by Sayedpiran et al., (2011) and Abdel‐Wareth and Lohakare, (2014). Different effects of supplemental mint on egg quality might stem from the amount and the source of mint extract.

### 4.3 Blood metabolites

No significant influence of experimental diets on albumin, uric acid, glucose, triglyceride and cholesterol was observed. There were some changes by addition of MEO, but the changes did not reach statistical significance. The results of this study revealed that LEO and MEO consumption could not change blood variables significantly. Most of spices and herbs enhance the synthesis and excretion of bile acids in the liver. As bile acids had beneficially effects on lipids’ digestion and absorption, it could improve the lipids’ digestion and absorption, which led to increase in the level of blood triglyceride (Srinivasan, 2005). Khursheed et al. mentioned that a variety of essential oil compounds, such as menthone, menthol and geraniol have been shown to suppress the hepatic 3‐hydroxy‐3‐methylglutaryl coenzyme A (HMG‐CoA) reductase activity (Khursheed et al., 2017). However, in the present study, the effect of the essential oils on blood triglyceride was not significant.

Changes in blood biochemical variables in the present experiment are not supported by other researchers (Nobakht & Mehmannavaz, 2010; Sayedpiran et al., 2011), who reported increase in feed intake and decrease in blood triglyceride level by adding 2% of *Lamiaceace menthapiperita*. In a research, mixture of three medicinal plants (0.5% *Malva silvestris*, 1% *Alhaji maurorum*, 0.5% *Mentha spicata*) decreased blood glucose of broilers (Nobakht & Shahryar, 2010). In addition, using 0.5% of *Mentha pulegium* powder significantly improved the performance and reduced the blood glucose of broilers (Nobakht et al., 2011).

Akbari and Torki reported that peppermint extract has increased total protein and HDL and decreased blood serum concentrations of total cholesterol, triglycerides, LDL and glucose of broilers (Akbari & Torki, 2014). Fallah, Kiani, & Azarfar also reported that peppermint extract (200 meg/kg) has increased albumin, HDL and significantly reduced total cholesterol, triglycerides, LDL and glucose in broilers (Fallah, Kiani, & Azarfar, 2013). It seems as some components of peppermint including menthol and menthone have a potential to decrease blood lipids in broilers (Escop (European Scientific Cooperative on Phytotherapy), 2003). Mansoub also reported that peppermint extract (0.75%, 1%, 1.5%, and 2%) increases albumin, total protein and HDL and also significantly decreases glucose, triglycerides, total cholesterol and LDL in serum of broilers; he also suggested that the main reason for the reduction of serum lipids in broilers is due to the active ingredients of peppermint leaves such as tocopherol and menthol (Mansoub, 2011). Our observation in this experiment on blood biochemical variables is not in agreement with Hardari, Nobakht, and Safamehr (2010) report, who stated that using 1.5% of *M. pulegium L*. had positive effects on blood cholesterol level.

Definitely, several variables such as supplementation methods (in diet or drinking water), the supplemental level, its level in the basal diet, bioavailability, stress condition, degree of stress, as well as duration of usage could be, at least in part, probable reasons for contradictory results in miscellaneous experiments.

## 5 CONCLUSION

Based on the results of the present study, it can be concluded that dietary supplemental MEO (250 mg/kg) may increase FCR, and LEO (250 mg/kg) is more effective than LEO (250 mg/kg) for egg production and egg mass purposes; besides MEO (250 mg/kg) negatively affected FCR compared with the control group.

## CONFLICT OF INTEREST

The authors of this manuscript have no conflicts of interest to declare.

## AUTHOR CONTRIBUTIONS

Mehran Torki: Conceptualization; Formal analysis; Funding acquisition; Investigation; Methodology; Project administration; Resources; Supervision; Validation; Writing‐review & editing. Ahmad Mohebbifar: Data curation; Software. Hamed Mohammadi: Validation; Writing‐original draft.

## ETHICAL STATEMENT

All the experimental proceedings in this experiment were approved by the Animal Ethics Committee of Razi University, Kermanshah, Iran.

### PEER REVIEW

The peer review history for this article is available at https://publons.com/publon/10.1002/vms3.343.
